# Sphingosine Kinase-1 Is Required for Toll Mediated β-Defensin 2 Induction in Human Oral Keratinocytes

**DOI:** 10.1371/journal.pone.0011512

**Published:** 2010-07-09

**Authors:** Manjunatha R. Benakanakere, Jiawei Zhao, Johnah C. Galicia, Michael Martin, Denis F. Kinane

**Affiliations:** 1 Department of Pathology, School of Dental Medicine, University of Pennsylvania, Philadelphia, Pennsylvania, United States of America; 2 School of Dentistry, Oral Health and Systemic Disease Center, University of Louisville, Louisville, Kentucky, United States of America; University Hospital Zurich, Switzerland

## Abstract

**Background:**

Host defense against invading pathogens is triggered by various receptors including toll-like receptors (TLRs). Activation of TLRs is a pivotal step in the initiation of innate, inflammatory, and antimicrobial defense mechanisms. Human β-defensin 2 (HBD-2) is a cationic antimicrobial peptide secreted upon Gram-negative bacterial perturbation in many cells. Stimulation of various TLRs has been shown to induce HBD-2 in oral keratinocytes, yet the underlying cellular mechanisms of this induction are poorly understood.

**Principal Findings:**

Here we demonstrate that HBD-2 induction is mediated by the Sphingosine kinase-1 (Sphk-1) and augmented by the inhibition of Glycogen Synthase Kinase-3β (GSK-3β) via the Phosphoinositide 3-kinase (PI3K) dependent pathway. HBD-2 secretion was dose dependently inhibited by a pharmacological inhibitor of Sphk-1. Interestingly, inhibition of GSK-3β by SB 216763 or by RNA interference, augmented HBD-2 induction. Overexpression of Sphk-1 with concomitant inhibition of GSK-3β enhanced the induction of β-defensin-2 in oral keratinocytes. Ectopic expression of constitutively active GSK-3β (S9A) abrogated HBD-2 whereas kinase inactive GSK-3β (R85A) induced higher amounts of HBD-2.

**Conclusions/Significance:**

These data implicate Sphk-1 in HBD-2 regulation in oral keratinocytes which also involves the activation of PI3K, AKT, GSK-3β and ERK 1/2. Thus we reveal the intricate relationship and pathways of toll-signaling molecules regulating HBD-2 which may have therapeutic potential.

## Introduction

Infection caused by sepsis is one of the leading causes of death in the United States [1], [2], [3]. Controlling inflammation from bacterial sepsis remains a challenge and antimicrobial peptides may have therapeutic utility [4]. Antimicrobial peptides, predominantly produced by epithelial linings, have shown broad spectrum activity against bacteria, fungi, viruses and parasites [5]. Defensins are potent cationic antimicrobial peptides present in mammals and insects [6], [7], [8], [9] consisting of two classes based on their structural characteristics, namely, α-defensins and β-defensins. Human α-defensins are found in granules of phagocytes and Paneth cells, whereas human β-defensins 2 (HBD-2) are highly expressed by epithelial cells [10], [11], [12]. Epithelial cells are a first line of defense against bacterial attack and thus understanding defensin induction mechanisms in these cells is crucial.

Sphingosine kinase-1 (Sphk-1) is an important intracellular enzyme that catalyzes a novel lipid messenger Sphingosine-1-phosphate (S1P) which regulates cellular proliferation and survival and histone acetylation [13], [14], [15] and activation implicated in cardio protection [16], [17], [18]. S1P is also a ligand for EDG1 (endothelial differentiation gene 1) receptor that regulates diverse cellular function [19]. Sphk-1 has been shown to regulate the MAPK signaling pathway and activates NF-kβ [20], and is highly expressed in various types of cancers [14] presumably associated with tumor angiogenesis. Recently, S1P has been shown to induce antimicrobial activity with both *in vitro* and *in vivo* animal infection models of *Mycobacterium tuberculosis* [21], [22], [23]. In the present investigation, we identified the involvement of Sphk-1 in the induction of β-defensin 2 in human gingival epithelial cells (HGECs) and also found that the inhibition of kinase glycogen synthase kinase-3β (GSK3-β) augments HBD-2, all of which may have therapeutic application.

## Methods

### Ethics statement

Gingival tissue biopsies were obtained with written informed consent from periodontally healthy patients undergoing crown-lengthening procedures at the University of Louisville's School of Dentistry with an Institutional Review Board approval. The gingiva was treated with 0.025% trypsin and 0.01% ethylenediaminetetraacetic acid overnight at 4°C and HGECs were isolated as previously described [24].

### Reagents

The cell culture medium was purchased from Invitrogen, CA. toll-like receptor (TLR) agonists namely, heat inactivated *P. gingivalis* was prepared as previously described [24], FSL-1 (Pam2CGDPKHPKSF), Pam3CSK4, *P. gingivalis* LPS, *E. Coli* LPS, ssRNA, Poly I:C, ODN 2006, Imiquimod, Flagellin were purchased from Invivogen, CA. Cell culture tested IL-1α, IL-1β and TNF-α cytokines were purchased from R&D Systems, S1P from Biomol International, PA. Sphk-1 (2-(*p*-Hydroxyanilino)-4-(*p*-chlorophenyl) thiazole) is a specific inhibitor for Sphk-1 that blocks the production of S1P), Wortmannin, U0126, LY 294002 and Akt inhibitors were purchased from EMD chemicals, NJ and SB 216763 was purchased from Tocris Bioscience, MO. Pre-validated siRNA specific to siSphk-1, siGSK-3β,control siRNA and transfection reagent siPORT NeoFX were purchased from Ambion, CA. Fugene 6 was purchased from Roche, IN. TransAM NF-kB p65 kit was purchased from Active Motif, CA and the ELISA kit for S1P was procured from Echelon Biosciences, UT, HBD-2 ELISA kit was from Peprotech, NJ and IL-6, IL-1β and TNF-α were from BD biosciences, CA. The antibodies phospho-Glycogen synthase (Ser641), phospho-p44/42 MAPK (ERK 1/2) and β-actin were purchased from Cell Signaling Technology, MA and phospho-Sphk-1 (ser225) was from ECM biosciences, KY.

### Cell culture and Challenge assays

HGECs were isolated and cultured as previously described [24], [25]. Briefly, when the cells were restored from frozen, they were subcultured and when reached confluence, they were pretreated for 2 h with 0.1 µM, 0.2 µM, 0.3 µM, 0.5 µM, 0.8 µM, 1.0 µM, 2.0 µM, 5.0 µM of Sphk-1 inhibitor and then challenged with FSL-1 (1 µg/ml) for 24 h. For TLR activation, heat inactivated *P. gingivalis* (MOI:100), FSL-1 (1 µg/ml), Pam3CSK4 (0.5 µg/ml), *P. gingivalis* LPS (1 µg/ml), *E. Coli* LPS (1 µg/ml), ssRNA (0.1 µg/ml), Poly I:C (5 µg/ml) ODN (0.5 µg/ml), Imiquimod (0.1 µg/ml), Flagellin (0.25 µg/mL), for IL-1R and TNF, IL-1α (2.5 ng/ml), IL-1β (2.5 ng/ml) and TNF-α (2.5 ng/ml) and S1P (100 nM) either in the presence or absence of Sphk-1 inhibition (2 µM). The challenge assay was performed for 24 h and culture supernatant was then collected. The secreted HBD-2 and cellular S1P was measured by ELISA. and NF-kB p65 was measured using TransAM NF-kB p65 kit in cells challenged with FSL-1 for 12 h after inhibiting Sphk-1 or GSK3.

### Real-time PCR

Total RNA was extracted from cultured cells by using TRIzol reagent (Invitrogen, Carlsbad, CA). The isolated total RNA samples were used to perform first strand cDNA synthesis (Applied Biosystems, Foster City, CA). Real-time PCR was performed by using 50 ng of cDNA with Sphk-1 (Assay ID: Hs00184211_m1), Sphk-2 (Hs00219999_m1) and GAPDH (Assay ID: 4333764F) as endogenous control as primers and probes on an ABI 7500 system (Applied Biosystems) in the presence of TaqMan DNA polymerase as previously described [26]. GAPDH was used as an endogenous control.

### Transfection

Primary epithelial cell cultures at the fourth passage were harvested, seeded at a density of 0.5×10^5^cells/well in a 6 well culture plate coated with type-I collagen, and maintained in 2 ml of medium until they reached 50–60% confluency. The epithelial cells were transfected with 100 pmol of siGSK-3β or siSphk-1 or non-target siRNA pool. Briefly, 100 pmol of siRNA was mixed with siPORT NeoFX Transfection Agent and incubated at room temperature for 15 min. The transfection mixture was then added drop wise to the respective wells and the reaction was incubated overnight. The following morning, the medium was replaced and cells were challenged as mentioned above. For pcDNA3-GSK3-β (S9A), pcDNA3-GSK3-β (K85A), pcDNA3 (empty vector control) and Sphk-1 overexpression, the plasmids were mixed with Fugene 6 transfection reagent and incubated for 15 min. The mixture was then added dropwise to the cells and transfection reaction was carried out for 24 h after which the cells were challenged as detailed above.

### Western blotting

The Western blots were performed by loading 50 µg total proteins on to each lane [26]. After electrophoresis, the membranes were incubated with antibody against phospho- Sphk-1 (Ser225) antibody, phospho-ERK p42/p44 antibody, phospho-Glycogen synthase (Ser641) and β-actin used as loading control. The membranes were developed using ECL plus™ western blotting detection reagent (GE Healthcare, Piscataway, NJ) and exposed using KODAK Imaging station 4000 MM with chemiluminescence detection. Brightness and contrast if needed were adjusted using powerpoint.

### Statistical analysis

The mRNA fold increase data was calculated according ΔΔCT method of Livak et al. [27]. Statistical analysis (analysis of variance and Tukey multiple comparison test) was done using GraphPad Pism 5.0 and GraphPad Instat 3.0. Statistical differences were considered significant at the p<0.05 level and indicated by an asterisk p<0.05 (*).

## Results

### Dose dependent inhibition of HBD-2 by Sphk-1 inhibition

**Figure 1:**
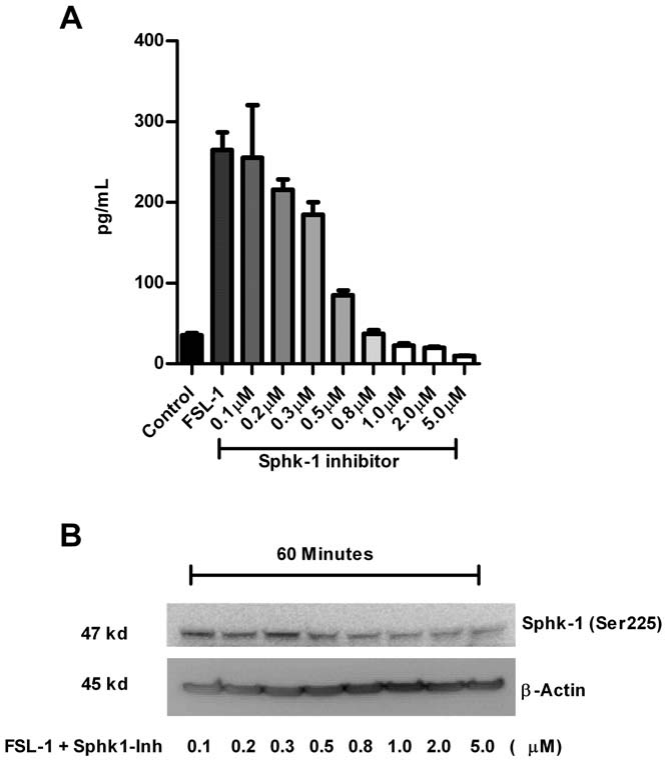
Dose dependent inhibition of HBD-2 induction in HGECs upon Sphk-1 inhibition. Keratinocytes were pretreated with Sphk-1 inhibitor at various concentrations ranging from 0.1 µM to 5.0 µM for 2 h prior to challenging the cells with FSL-1 ligand (1 µg/ml) for 24. Supernatant was subjected to human HBD-2 ELISA. Sphk-1 inhibitor dose dependently inhibited the induction of HBD-2 (**A**). In another set of experiment, total protein was collected after 60 min of challenge as mentioned above. Immunoblot was performed with ser225 phospho specific Sphk-1 antibody and β-actin as loading control. The level of phosphorylation of Sphk-1 at ser225 was dose dependently down regulated upon Sphk-1 inhibitor treatment (**B**). Results are mean ± SEM and are representative of three independent experiments.

Sphk-1 catalyzes a novel lipid messenger S1P [13]. S1P regulates diverse physiological and pathological processes including cancer and inflammation involving proliferation, migration, invasion, and angiogenesis *in vitro* and in animals [14], [28], [29]. HBD-2 is upregulated in various cancers [30], [31], [32], [33]. Based on these findings, we hypothesized that a direct link between the upregulation of Sphk-1, S1P and the induction of HBD-2 in oral keratinocytes. To address our hypothesis, we utilized the TLR2 ligand (FSL-1) to challenge HGECs as TLR2 has been shown to be an important receptor in gingival epithelial cell innate immune responses [26], [34]. First we pretreated primary HGECs with various concentrations of Sphk-1 inhibitor for 2 h followed by stimulating the cells with FSL-1 to determine the optimal dose. After 24 h of challenge, we measured HBD-2 induction by ELISA. The inhibition of Sphk-1 by a pharmacological inhibitor dose dependently inhibited the induction of HBD-2 in HGECs (Fig. 1A). We also wanted to check the phosphorylation state of Sphk-1 in the presence of Sphk-1 inhibitor to verify its kinase activity because the phosphorylation of Sphk-1 at Ser225 has been shown to elevate its kinase activity by increasing S1P production [20]. The total protein from the 60 min challenge with FSL-1 in the presence of different concentration of Sphk-1 inhibitor was subjected to immunoblot against phospho-specific Sphk-1 (Ser225). The inhibition revealed a dose dependent decrease in the level of phosphorylation of Sphk-1 at serine 225 (Fig. 1B). The phosphorylation of Sphk-1 at serine 225 by ERK 1/2 is critical for the activation of NF-kB by S1P [20]. Hence a decrease in the phophorylation level of Sphk-1 may account for decreased NF-kB activity limiting HBD-2 mRNA transcriptional activation: as NF-kB has been shown to be a critical transcription factor in the induction of HBD-2 [35].

### Inhibition of Sphk-1 abrogates HBD2 induction by keratinocytes

**Figure 2:**
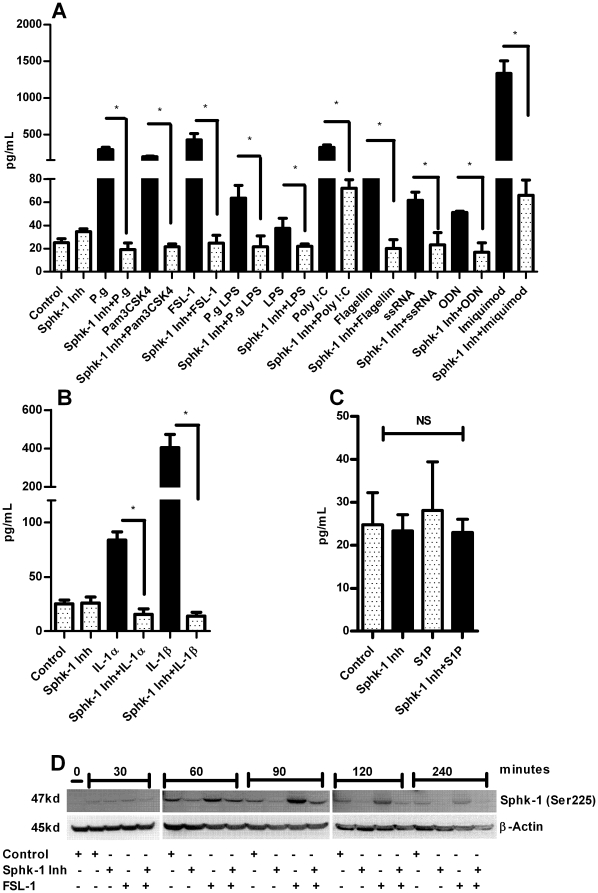
Sphk-1 inhibition down modulates agonists induced HBD-2. Oral keratinocytes were incubated with or without Sphk-1 inhibitor (2 µM) for 2 h prior to challenging the cells with various TLR agonists namely, heat inactivated *P. gingivalis* (MOI:100), FSL-1 (1 µg/ml), Pam3CSK4 (0.5 µg/ml), *P.gingivalis* LPS (1 µg/ml), *E. Coli* LPS (1 µg/ml), ssRNA (0.1 µg/ml), Poly I:C (5 µg/ml) ODN (0.5 µg/ml), Imiquimod (0.1 µg/ml), Flagellin (0.25 µg/mL) (**A**); the IL-1α (2.5 ng/ml), IL-1β (2.5 ng/ml) and TNF-α (2.5 ng/ml) (**B**) and GPCR agonist S1P (100 nM) (**C**) for 24 h. The supernatant was collected after 24 h and HBD-2 ELISA was performed using Human BD-2 ELISA kit. The Sphk-1 inhibitor ablated HBD-2 induction with the agonists tested. The time course experiment was performed by pretreating the cells with Sphk-1 (2 µg/ml) for 2 h before challenging with FSL-1 (1 µg/ml) for 0, 30, 60, 90, 120 and 240 min. The total protein was collected and subjected to immunoblot with ser225 phospho specific Sphk-1 antibody and β-actin as loading control. We noted increase in the phosphorylation level of Sphk-1 at ser225 as early as 60 min and the level of phosphorylation was down regulated in the presence of Sphk-1 inhibitor (**D**). Control cells received DMSO unless otherwise stated. Results are mean ± SEM and are representative of three independent experiments. Statistical comparisons are shown by horizontal bars with asterisks above them (* indicates p<0.05 determined by ANOVA and Tukey multiple comparison test).

**Figure 3:**
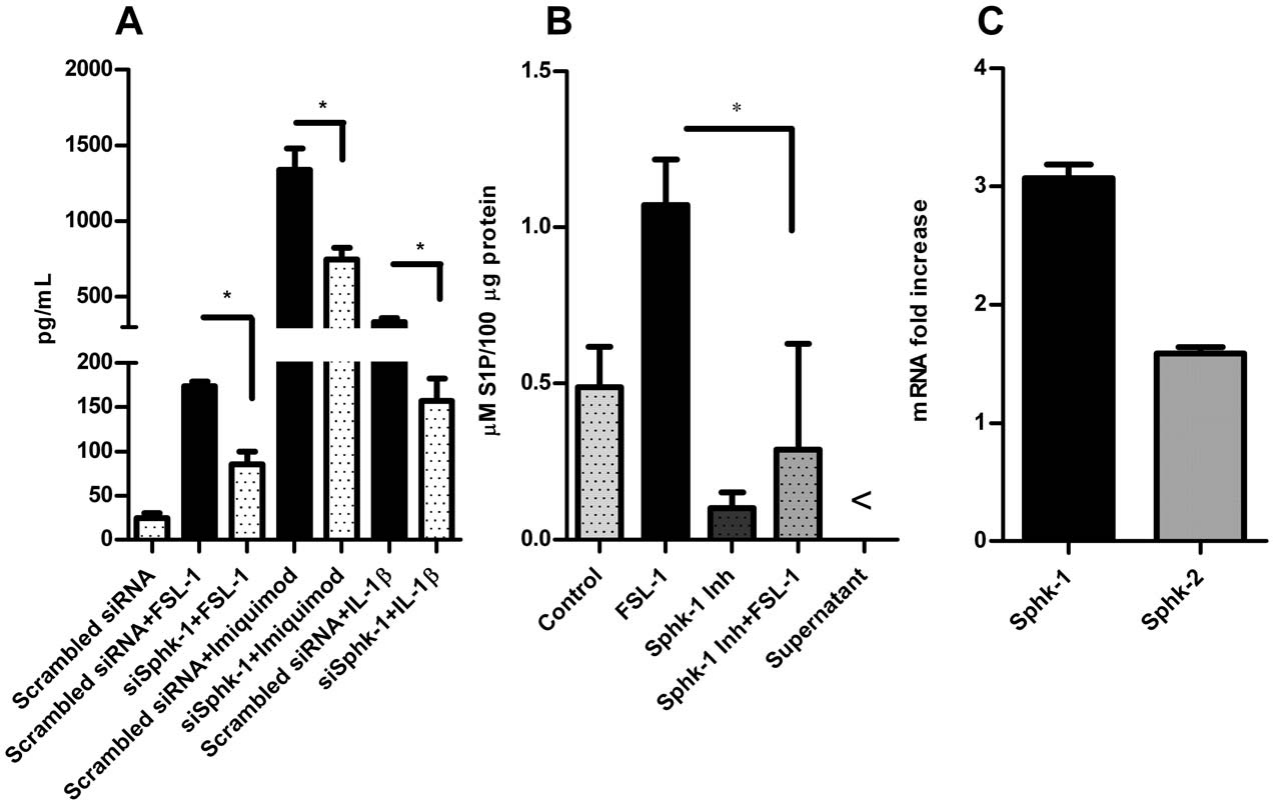
RNAi mediated inhibition of Sphk-1 inhibits agonists induced HBD-2. The cells were transiently transfected with siRNA against Sphk-1 and incubated with FSL-1 (1 µg/ml), Imiquimod (0.1 µg/ml) and IL-1β (2.5 ng/ml). The supernatant was collected after 24 h challenge and subjected to human HBD-2 ELISA. siRNA against Sphk-1 down modulated agonist induced HBD-2 in HGECs (**A**). Intracellular S1P was measured using S1P ELISA kit after challenging with FSL-1 (1 µg/ml) for 2 h in cells pre-incubated with Sphk-1 inhibitor. The reaction was terminated after 2 h and 100 µg of total protein was subjected to ELISA. The intracellular S1P production was downregulated by Sphk-1 inhibitor (**B**). Total RNA was collected and converted to cDNA from the cells challenged with FSL-1 (1 µg/ml) for 24 h. cDNA was subjected to real time PCR with Sphk-1, Sphk-2 and GAPDH endogenous control TaqMan probes. Sphk-1 mRNA expression was highly upregulated compared to Sphk2 mRNA expression showing Sphk-1 as a predominant kinase in HGECs upon ligand challenge (**C**). Control cells received DMSO unless otherwise stated. Results are mean ± SEM and are representative of three independent experiments. Statistical comparisons are shown by horizontal bars with asterisks above them (* indicates p<0.05 determined by ANOVA and Tukey multiple comparison test).

HBD-2 can be induced by the activation of various TLRs and possess strong antibacterial activity against Gram-negative bacteria [36], [37], [38]. In order to see the effect of our Sphk-1 inhibition finding, we challenged HGECs with various TLR ligands either in the presence or absence of Sphk1 inhibiton for TLR2 (heat killed *P. gingivalis*, *P. gingivalis* LPS, Pam3CSK4, FSL-1) TLR3 (Poly I:C), TLR4 (*E. coli* LPS), TLR5 (Flagellin), TLR7 (ssRNA & Imiquimod), TLR9 (ODN) after determining the optimal doses for each of the agonists. Surprisingly, HBD-2 induction was significantly down-regulated in the presence of Sphk-1 inhibitor in cells treated with TLR ligands (Fig. 2A). This inhibition demonstrates that Sphk-1 activation is crucial in TLR mediated induction of HBD-2. It has already been shown that HBD-2 can be strongly induced by exogenous TNFα and IL-1β cytokines [35]. To address this issue and to check the effect of Sphk-1 inhibition on TNF and IL-1 receptors, we challenged the cells with IL-1α, IL-1β and TNFα in the presence or absence of Sphk-1 inhibition. We also utilized S1P, a ligand for the S1P_1_ receptor [19] in the presence or absence of the Sphk-1 inhibitor (sphingosine is converted to S1P by sphingosine kinase) [29]. Sphk-1 inhibitor strongly inhibited the induction of HBD-2 in cells treated with cytokines but not with the addition of extracellular S1P (Fig. 2B & C). Next, we performed a time course experiment for determining the phosphorylation state of Sphk-1 in the presence of inhibitor. The phosphorylation of Sphk-1 at Ser225 started as early as 30 min and reached its maximum at 90 min after addition of FSL-1 to the cells. However, the pharmacological inhibition of Sphk-1 down regulated its kinase activity by down regulating phosphorylation on Ser225 (Fig. 2D). We wanted to examine whether silencing of Sphk-1 mRNA with siRNA against Sphk-1 has the same effect as the pharmacological inhibition. HBD-2 induction was measured in HGECs transfected either with siRNA against Sphk-1 or non targeting siRNA, 24 h after challenge with FSL-1, Imiquimod and IL-1β. RNA silencing also revealed significant down regulation of HBD-2 in the cells transfected with siRNA against Sphk-1 confirming the results from pharmacological inhibition (Fig. 3A). We noted that the exogenous addition of S1P to the cells failed to induce HBD-2, in contrast to the data obtained in a mouse model [23], so we were compelled to examine the intracellular S1P levels both in the challenged state and also with inhibitor. Indeed, the agonist FSL-1 induced significantly higher intracellular S1P, an increase which was abrogated in the presence of Sphk-1 inhibitor (Fig. 3B) and correlated with the phosphorylation state of Sphk-1. Further, we wanted to test if Sphk-2 contributed to the increase of intracellular S1P levels in HGECs after challenging with FSL-1. Thus we performed real time PCR to determine the mRNA expression levels of both Sphk-1 and Sphk-2 in the challenged state. Sphk-1 mRNA expression was significantly upregulated compared to Sphk2 upon FSL-1 challenge (Fig. 3C). This data demonstrates that Sphk-1 is the predominant sphingosine kinase involved in the intracellular conversion of S1P in HGECs. These results clearly show intracellular S1P plays an important role in the induction of HBD-2 and also underline the importance of phosphorylated Sphk-1 at Ser225 in converting S1P when TLRs are triggered.

### GSK-3β inhibition augments HBD-2 in oral keratinocytes

**Figure 4:**
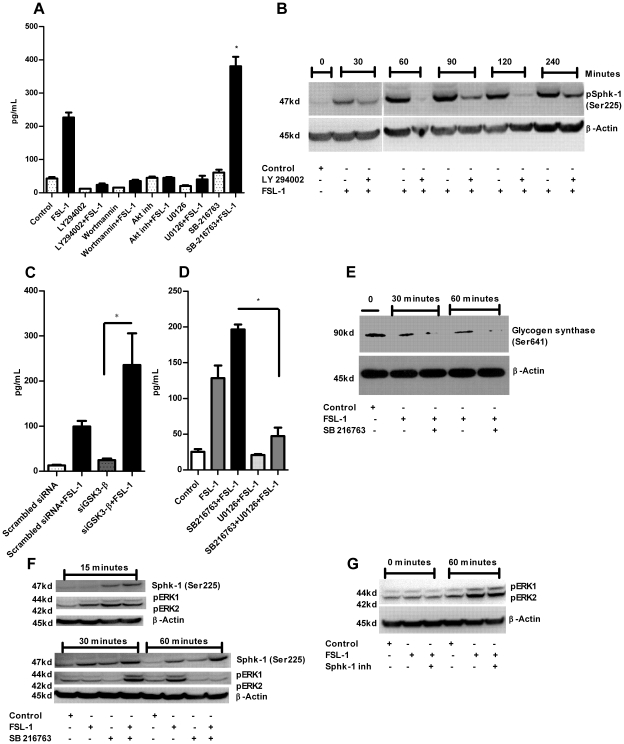
Inhibition of GSK3 augments HBD-2 induction. Keratinocytes were pretreated with PI3K inhibitor (LY294002–10 µM and wortmannin –0.5 µM) or Akt inhibitor (10 µM) or MEK 1/2, U0126 (25 uM) or GSK3 inhibitor (SB216763-12 µM) 2 h before challenging with FSL-1(1 µg/ml) for 24 h. The supernatant was subjected to human HBD-2 ELISA. PI3K, Akt and MEK inhibitors abrogated HBD-2 induction whereas GSK3 inhibitor augmented HBD-2 induction in HGECs (**A**). The cells were pretreated with LY294002 (10 µM) for 2 h and challenged with FSL-1 (1 µg/ml) for 0, 30, 60, 90, 120 and 240 min. Total protein was subjected to immunoblot against phospho- ser225 Sphk-1 antibody with β-actin as loading control. PI3K inhibitor downregulated the phosphorylation level of Sphk-1 at Ser225 demonstrating the pivotal role of PI3K in Sphk-1 activation (**B**). The cells were transient transfected of siRNA against GSK-3β and challenged with FSL-1 (1 µg/ml) for 24 h and supernatants were subjected to human HBD-2 ELISA. siGSK3-β up regulated HBD-2 induction after challenging with FSL-1. This upregulation of HBD-2 was significantly higher to FSL-1 challenge alone (**C**). The cells were either pretreated with U0126 (25 uM) and/or SB216763 (12 µM) prior to challenging with FSL-1 (1 µg/ml) for 24 h and supernatant was subjected to HBD-2 ELISA. GSK3 inhibitor augmented the HBD-2 induction whereas the cells with GSK3 +MEK 1/2 inhibitor ablated HBD-2 induction (**D**). Phospho-Glycogen synthase (Ser641) levels were assessed by incubating SB216763 (12 µM) for 2 h prior to challenge with FSL-1 for 0, 30 and 60 min. Total protein was subjected to immunoblot against Phospho-Glycogen synthase (Ser641) antibody with β-actin as loading control. The phosphorylation of Glycogen synthase at Ser641 was down regulated in the presence of SB216763 (**E**). Time course experiment was performed either in the presence or absence of SB 216763 inhibitor (GSK3) (12 µM). The cells were challenged with FSL-1 (1 µg/ml) and the total protein was collected at 15, 30 and 60 min and subjected to immunoblot against phospho- ser225 Sphk-1 antibody and p44/42 MAPK (Erk1/2) antibody with β-actin as loading control. Inhibition of GSK3 by SB 216763 increased the Sphk-1 phospho-ser225 at 60 min, ERK 1/2 phosphorylation increased at 30 min of agonist challenge (**F**). The phosphorylation of p44/42 MAPK (Erk1/2) was unaltered upon Sphk-1 inhibitor after 60 min demonstrating Sphk-1 downstream of Erk 1/2 (**G**). Control cells received DMSO unless otherwise stated. Results are mean ± SEM and are representative of three independent experiments. Statistical comparisons are shown by horizontal bars with asterisks above them (* indicates p<0.05 determined by ANOVA and Tukey multiple comparison test).

GSK3β is one of the major kinases involved in TLR signaling and is a serine threonine kinase regulating various cellular processes [39], [40]. GSK3 can be inhibited by phospho-Akt, a member of the PI3K pathway [41]. Its inhibition by lithium or SB 216763 (pharmacological inhibitor) increased the anti-inflammatory cytokine IL-10 by driving the cells from the inflammatory pathway towards an anti-inflammatory pathway [41]. In A549 cells, PI3K has been shown to regulate HBD-2 secretion [35] and recently an antimicrobial peptide LL-37 has been shown to be upregulated by increased PI3K activity and the Akt pathway, suppressing GSK3 phosphorylation in HaCat cell lines [42]. Moreover, HBD-2 has also been shown to have anti-inflammatory properties like IL-10 [43]. Since GSK-3β is regulated by PI3K [41], we wanted to test whether the inhibitors against PI3K, AKT, MEK 1/2 and GSK3 influence HBD-2 induction in HGECs. We first pretreated HGECs with LY 294002 and Wortmannin (PI3K inhibitors), AKT inhibitor, U0126 (MEK inhibitor) and SB-216763 (GSK3 inhibitor) for 2 h followed by FSL-1 treatment for 24 h prior to measuring HBD-2 induction. The inhibitors of PI3K, AKT and U0126 abrogated the secretion of HBD-2 whereas GSK3 inhibitor induced significantly higher amounts of HBD-2 compared to FSL-1 alone. This data demonstrated that inhibition of GSK3 by SB 216763 augmented HBD-2 secretion via the PI3K dependent pathway (Fig. 4A). Our data on PI3K activity is in agreement with the HBD-2 induction reported in A549 cells [35]. We also wanted to test whether Sphk-1 is up or downstream of PI3K. If Sphk-1 is downstream of PI3K, the pharmacological inhibition of PI3K should down regulate Sphk-1 activity. To demonstrate this, the cells were pretreated with PI3K inhibitor LY294002 for 2 h and challenged with FSL-1 for 0, 30, 60, 90, 120 and 240 min. The total protein was collected and subjected to immunoblot against Sphk-1 phospho-specific antibody (Ser225). The immunoblot data clearly shows PI3K inhibition directly blocks the activity of Sphk-1 by decreasing the phosphorylation of Sphk-1 at Ser225 (Fig. 4B) and confirms that the Sphk-1 is downstream of PI3K in HGECs.

The findings from the pharmacological inhibition of GSK3 compelled us to confirm if RNA silencing of GSK3-β will have similar effect to that of the inhibitor SB 216763. To address this, we transfected HGECs with siRNA against GSK3-β and challenged the cells with FSL-1 for 24 h. The silencing of GSK-3β significantly increased the induction of HBD-2 in the presence of FSL-1 demonstrating that the inhibition of GSK3-β either by siRNA or by pharmacological inhibition can augment HBD-2 induction in HGECs (Fig. 4C). Interestingly, we could not detect IL-10 in the culture supernatants of HGECs even after 96 h of post challenge with *E. coli* LPS, FSL-1, PAM3CSK4, Poly I:C or with heat inactivated *P. gingivalis* in the presence or absence of GSK3 inhibition.

The involvement of GSK-3β in regulating ERK 1/2 activation was assessed by pretreating the cells with U0126 and/or SB216763 before challenging with FSL-1 for 24 h. HBD-2 induction was dramatically upregulated in the presence of SB216763 and FSL-1. This upregulation was ablated in the presence of U0126 and SB216763 together (Fig. 4D). This data is in agreement with Rehani et al. (2008) who show that ERK 1/2 activation is mediated by the inhibition of GSK-3β [44]. We further investigated if SB216763 blocks GSK3 and functionally thus Glycogen synthase activity. To address this, the cells were pretreated with SB216763 and challenged with FSL-1 for 0, 30 and 60 min. The activity of Glycogen synthase was assessed by immunoblotting against phospho-Glycogen synthase (Ser641) antibody. The phosphorylation level of Glycogen synthase at Ser641 was significantly down regulated in the presence of SB216763 and FSL-1 demonstrating the specificity of GSK3 inhibition (Fig. 4E). Interestingly, the phophorylation levels of Sphk-1 at ser225 and ERK p42/p44 increased upon GSK3 inhibition (Fig. 4F). The increase in Sphk-1 might be due to the increase in ERK phosphorylation and its ability to phosphorylate Sphk-1 at Ser225 as observed earlier [20]. We also tested if Sphk-1 inhibition had any effect on ERK 1/2 activity. The inhibition of Sphk-1 showed no significant effect on ERK 1/2 activity as revealed by its phosphorylation at 60 min post challenge with Sphk-1 inhibition (Fig. 4G). This data demonstrates that Sphk-1 is a downstream kinase to ERK 1/2 in HGECs.

### GSK3-β over-expression downregulated HBD-2 induction

**Figure 5:**
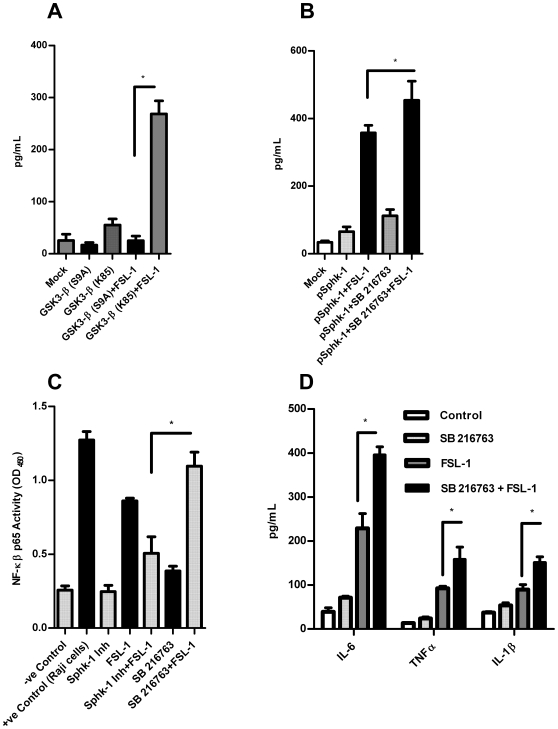
Overexpression of GSK3-β in HGECs. Cells were transiently transfected with GSK3-β (S9A), GSK3-β (K85) and pCDNA3.1 as Mock using Fugene 6. The transfected cells were challenged with FSL-1 (1 µg/ml) for 24 h. The supernatant was subjected to human HBD-2 ELISA. The cells transfected with kinase dead GSK-3β (K85) plasmid induced significantly higher amounts of HBD-2 however; the cells transfected with constitutively active GSK3-β (S9A) attenuated HBD-2 induction (**A**). We overexpressed Sphk-1 by transfecting the cells with Sphk-1 plasmid and incubated in the presence or absence of GSK3-β inhibitor SB 216763. The FSL-1 (1 µg/ml) challenged cells induced significantly higher amounts of HBD-2 compared to the cells without GSK3 inhibition (**B**). NF-kB p65 activity was measured in the presence or absence of Sphk-1 and GSK3 inhibitor. Sphk-1 inhibition reduced NF-kβ activity however, NF-kβ activity increased upon GSK3 inhibition when challenged with FSL-1 (1 µg/ml) (**C**). The cells were pretreated with SB216763 for 2 h prior to challenging with FSL-1 for 24 h and supernatant was subjected to ELISA using appropriate kits. IL-1β, TNFα and IL-6 were significantly upregulated upon GSK3 inhibition in the presence of FSL-1 (**D**). Control cells received DMSO unless otherwise stated. Results are mean ± SEM of triplicates and are representative of three independent experiments. Statistical comparisons are shown by horizontal bars with asterisks above them (* indicates p<0.05 determined by ANOVA and Tukey multiple comparison test).

Since we noted dramatic upregulation of HBD-2 following inhibition of GSK3 by a potent selective inhibitor SB 216763 and also by siRNA, we tested if the overexpression of GSK3-β downregulates HBD-2. Thus HGECs were transfected with plasmids carrying GSK3-β (K85A) expressing ‘kinase dead’ [45] (that lack kinase activity) and GSK3-β (S9A) mutated plasmid to see the effect on constitutively active GSK3-β and the contribution of serine 9 phosphorylation to the induction of HBD-2. After 24 h of transfection, the cells were challenged with FSL-1 for 24 h and HBD-2 levels were measured in the supernatants. When we over-expressed the constitutively active GSK3-β (S9A) followed by FSL-1 stimulation, the induction of HBD-2 was attenuated. However, when kinase dead GSK3-β (K85) was over-expressed, the HBD-2 induction was dramatically increased following FSL-1 challenge demonstrating GSK3-β strongly modulates HBD-2 induction in HGECs (Fig. 5A).

### Over-expression of Sphk-1 enhances HBD-2 induction

Sphk-1 has been shown to possess anti-apoptotic activity [46], in part due to increased intracellular S1P levels, because S1P regulates cellular proliferation [15]. Interestingly, HBD-2 levels have been shown to be higher in proliferating cells [47]. Since we noted the abrogation of HBD-2 by Sphk-1 inhibitor, we wanted to overexpress Sphk-1 in HGECs to check if it contributes to increase in the induction HBD-2. To demonstrate this, we over-expressed the Sphk-1 in HGECs by transient transfection. After 24 h of transfection, the cells were cultured with FSL-1 - for 24 h. The overexpression of Sphk-1 in HGECs markedly increased HBD-2 levels after FSL-1 challenge (Fig. 5B) confirming our hypothesis. Further, we also wanted to see the effect of HBD-2 induction by concomitant inhibition of GSK3 in cells overexpressing Sphk-1. First, we transfected the cells with Sphk-1 plasmid and at 24 h post transfection we pretreated the cells with SB 216763 for 2 h followed by FSL-1 treatment for 24 h. After 24 h of challenge, we noted robust increases in the induction of HBD-2 upon GSK3 inhibition in cells over-expressing Sphk-1. This data clearly demonstrates the pivotal roles of Sphk-1 and GSK3-β in the induction of HBD-2 (Fig. 5B).

We further investigated NF-kB activity by measuring p65 in cells pretreated with Sphk-1 inhibitor to check whether Sphk-1 inhibition caused NF-kB inactivation leading to down-regulated HBD-2 induction. The Sphk-1 inhibitor reduced NF-kB activity in HGECs upon FSL-1 treatment (but did not result in complete inhibition at the 2 µM concentration). This result is in agreement with the previously observed NF-kB activation following blocking of Sphk1 by anti-sense oligo [48]. Intriguingly, the inhibition of GSK3 by SB 216763 strongly upregulated NF-kB activity (Fig. 5C). The upregulation of NF-kB in the inhibition of GSK3 by SB 216763 shows that the kinase activity of GSK3 functions differently in non-myeloid cells and does not modulate IL-10 expression as it does in immune cells [41]. However, the mechanism of this increase in NF-kB activity upon inhibition of GSK3 in epithelial cells has yet to be explored. Since we noted upregulation of both ERK 1/2 and NF-kB on GSK-3 inhibition in HGECs, we wanted to see if there was any difference in proinflammatory cytokine induction. Interestingly, the induction of proinflammatory cytokines (IL-6, TNFα and IL-1β) was significantly upregulated upon GSK3 inhibition by SB 216763 (Fig. 5D). This is in marked contrast to immune cells where GSK3 inhibition down regulated proinflammatory cytokines [41]. Further investigation of the transcription factor CREB and its interaction with CBP in HGECs might uncover the mechanism for the different effects of GSK3 in non-myeloid cells.

## Discussion

Microbial elimination is a critical step in controlling the onset of chronic inflammation. Although this can be achieved by antibiotic treatment, development of microbial resistance is increasingly limiting antibiotic utility [49]. For this reason, a number of small molecules have been identified that may function as antimicrobial agents [50]. Short cationic peptides produced by cells with antibacterial and/or immunomodulatory activity are being heralded as novel approaches in the control of bacterial pathogenesis [51]. HBD-2 is an antimicrobial peptide that can be strongly induced by various microbial products. Stimulation of TLRs by its ligands results in the recruitment of an adaptor molecule, Myd88, which activates a downstream signaling pathway that includes interleukin 1 receptor associated kinase (IRAK) [52], which in turn activates NF-kB, p38 and JNK pathways that lead to proinflammatory cytokine and antimicrobial peptide secretion. Other than antimicrobial activity, HBD-2 has been shown to possess chemotactic properties, induce cytokines, promote DC maturation and antigen-specific B and T cell responses [53]. HBD-2 is primarily potent against Gram-negative bacteria [53] and disrupts bacterial membranes by electrostatic interaction between the HBD-2 and the negatively charged phospholipids on the bacteria or by formation of ion channels by defensin oligomers, whilst also blocking pathogen entry into host cells and inhibiting pathogen replication [53], [54], [55], [56]. There are several reports on the regulation and secretion of HBD-2 in various types of cells [57], [58], [59], [60], [61], [62]. However, the mechanism of HBD-2 induction by various microbial products that elicit antimicrobial defense through TLRs in epithelial cells has not been elucidated. Epithelial cell signaling is crucial in early defense against microbes as is HBD-2 regulation and secretion, in the recognition and clearance of bacteria in chronic inflammatory diseases such as periodontitis. Enhancing the production of epithelial HBD-2 to combat the pathogen attack is a valuable approach against sepsis.

**Figure 6:**
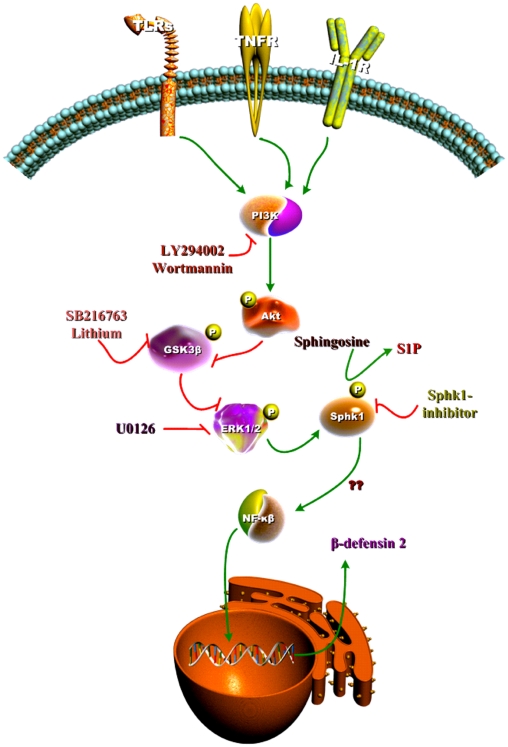
Model of mechanism involved in HBD-2 secretion in gingival epithelial cells. Triggering TLRs by respective ligand stimulates the cells to induce HBD-2. This induction can be ablated by a pharmacological inhibitor against Sphk-1, and inhibition of GSK3 by SB216763 or siGSK3-β in the absence of Sphk-1 inhibition can augment HBD-2 secretion. Inhibition of PI3K by either wortmannin or LY294002 abrogated HBD-2 in gingival epithelial cells. PI3K activated Akt, the phosphorylation of Akt inhibited GSK3 in turn activating ERK 1/2. ERK 1/2 activates Sphk-1 by phosphorylation at Ser225 and increase NF-kB activity. This show PI3K-Akt-GSK3-ERK1/2-Sphk-1 mediates HBD-2 synthesis in gingival epithelial cells.

It has recently been shown that the gene specific chromatin remodeling, accounts for transient silencing of proinflammatory cytokine genes and promoting antimicrobial gene activation [63]. Here we report for the first time the involvement of a novel kinase, Sphk-1 in the induction of the antimicrobial peptide HBD-2 that is augmented by suppressing GSK3-β in human oral keratinocytes independent of proinflammatory cytokine induction that involve TLRs-PI3K-Akt-GSK3-ERK1/2-Sphk-1 (Fig. 6). Moreover, we also show that the activation of various TLRs and cytokines such as IL-1α/β and TNFα can induce HBD-2, and Sphk-1 inhibition can ablate this induction. S1P has been shown to induce antimicrobial activity in a mouse model of *Mycobacterium tuberculosis* infection, both *in vivo* and *in vitro* [21], [22], [23]. The host's innate immune response to *M. tuberculosis* is mainly elicited by TLR2 [64]. However, the mechanism of antimicrobial activity in these cases has not been explored. We have previously shown that S1P in combination with LPS cooperate in the induction of proinflammatory cytokines and the inhibition of Sphk-1 did not affect the induction of proinflammatory cytokines in HGECs [65]. Here we demonstrate that the Sphk-1 inhibitor dose dependently down-regulated HBD-2 induction through all the TLR ligands and cytokines tested. Additionally, we show that Sphk-1 is predominantly activated compared to its isoform Sphk-2 by a TLR2 ligand in HGECs. In order to confirm and extend the mechanism behind this, we carried out experiments using TLR2 as a representative receptor because TLR2 has been implicated as an important receptor in the human gingival keratinocyte innate immune response [26] and is required for innate responses to *P. gingivalis* [66] and its clearance *in vivo* [67] and also has been shown to activate PI3K [68]. It is well known that PI3K can mediate activation of various TLRs [69] hence it is plausible to focus on TLR2 as a model receptor to dissect HBD-2 induction pathway by TLRs in HGECs. Interestingly, TLR2 stimulation enhanced Sphk-1 activation by increasing its phosphorylation at Ser225. Phosphorylation events of Sphk-1 are dependent on ERK 1/2 [20]. The only phosphorylation site of Sphk-1 at Ser225 is involved not only in its catalytic activity but is also important in its translocation to the plasma membrane [29] where sphingosine is converted to S1P and this conversion leads to cell proliferation and survival which is supported by over-expression of kinase inactive enzyme [70]. In conjunction, HBD-2 has been shown to be elevated in proliferating cells [47]. Our data on phosphorylation of Sphk-1 is in agreement with the above wherein PI3K and MEK inhibitor suppressed the agonist activated phosphorylation of Sphk-1 as well as the induction of HBD-2. Moreover, Sphk-1 inhibition did not affect the activation of ERK 1/2 in HGECs. Interestingly, HBD-2 activity is also increased in some viral infections such as activity against RSV infection [71], viral haemorrhagic septicaemia rhabdovirus [72], HIV-I [73], and papilloma virus [74]. Moreover, the activity of Sphk-1 has been shown to be increased in viral infections [75]. Compatible with the above, inhibition of Sphk-1 has been shown to increase NS3 viral replication in bovine viral diarrhea [75]. Perhaps, the inhibition of Sphk-1 may result in down regulation of antimicrobial peptide synthesis which might have affected enhanced viral replication. Hence increasing HBD-2 by modulating the kinase activity of Sphk-1 and kinase inactivation of GSK3-β can be seen as a valuable intervention against viral attack.

GSK3 is part of the PI3K signaling network and is also one of the major kinases regulating TLR signaling. Inhibition of GSK3 by lithium or SB 216763 (pharmacological inhibitor) or by siRNA increased the anti-inflammatory cytokine IL-10 by driving the immune cells from an inflammatory to an anti-inflammatory pathway [41]. S1P has also been reported to induce anti-inflammatory activity, which is achieved by sequestering lymphocytes in lymph nodes [76]. To our surprise, the inhibition of GSK3-β augmented both HBD-2 and proinflammatory cytokines like IL-6, TNFα and IL-1β. In support of this, transient silencing of GSK3β up-regulated agonist induced HBD-2. Further, the agonist induced HBD-2 was down-regulated by ERK 1/2 inhibition. In addition inhibition of both GSK3 and ERK 1/2 at the same time down-regulated HBD-2. This indicates that the inhibition of GSK3 enhances ERK 1/2 activation. To further verify whether and if GSK3 inhibition is specific, the phosphorylation of glycogen-synthase kinase (a downstream kinase of GSK3) was assessed. The phosphorylation level of glycogen-synthase (Ser641) was markedly down regulated with SB 216763 treatment demonstrating the efficacy of GSK3 inhibition. The inhibitory role of GSK3-β was further verified by over-expression of kinase dead GSK3-β. In this case constitutively active GSK3β (S9A) inhibited HBD-2 possibly blocking the activation of ERK 1/2. But the secretion of HBD-2 was restored by kinase dead GSK3-β over-expression. Concomitant overexpression and suppression of Sphk-1 and GSK3 respectively, yielded higher HBD-2 induction.

Our present findings demonstrate that Sphk-1 regulates HBD-2 induction by TLRs at least in human oral epithelial cells. The suppression of HBD-2 by pharmacological inhibition of Sphk-1 was further confirmed with RNAi. We have also experimentally shown the increase in Sphk-1 activity by TLR activation contributes to an increase in the intracellular S1P. In contrast to intracellular S1P data, extracellular addition of S1P failed to up-regulate HBD-2 in our model. Activation of S1P_1_ by extracellular S1P may require TLR cooperation as demonstrated earlier [65]. However, further experimental evidence is needed to confirm the role of extracellular activation of S1P_1_ in gingival keratinocytes. The increase in Sphk-1 activity may be due to the up regulated ERK 1/2 phosphorylation and agonist induced GSK3-β inhibition. Additionally, PI3K involvement in increasing ERK 1/2 phosphorylation by inhibition of GSK3 has been shown previously [77], [78]. This increase might be due to the suppression of phospho-RAC1 at Ser71 and subsequent phosphorylation of PAK1 at Ser199/204 and cRaf at Ser338 activating ERK 1/2 as observed in human monocytes by Rehani et al. (2008) [44] or due to the activation of PKCδ as observed in human cell lines HT29 and Caco-2 by Wang et al. (2006) [79]. In contrast to the above findings, we observed increased NF-kB activity upon GSK3 inhibition. This show GSK3 negatively or positively affects either ERK 1/2 and/or NF-kB in a cell type specific manner. Further understanding of the involvement of different transcription factors will answer basic mechanistic questions involved in HBD-2 induction. In HGECs, ligand induced suppression of GSK3-β may induce chromatin modifications of both proinflammatory cytokine and antimicrobial peptide genes that result in higher gene activation. Activation of proinflammatory genes after GSK3 inhibition may be exclusive to non-myeloid cells such as HGECs as similar experiments with immune cells show decreased proinflammatory gene activation [41]. Moreover, the absence of IL-10 calls into question the ability of epithelial cells to dampen the inflammation caused by bacteria or viruses. Epithelium may have been evolved to induce proinflammatory cytokines and chemokines to recruit immune cells to the site of infection in lieu of secreting anti-inflammatory cytokine such as IL-10 to dampen the inflammation on its own. However it can induce antimicrobial peptides to combat against invading microbes.

The present investigation identifies: i) Sphk-1 as a critical kinase involved in TLR mediated human β-defensin 2 induction and modulation in HGECs; ii) activation of TLRs induces phosphorylation of Sphk-1 and its intracellular activity by enhanced conversion of S1P; and iii) activated PI3K suppresses GSK3-β augmenting HBD-2 induction. Collectively, it is plausible to hypothesize that the modulation of Sphk-1 and GSK3-β to induce and enhance HBD-2 production may help cells combat bacterial insult and thus they have potential therapeutic value.
